# Congenital hepatic fibrosis leading to cirrhosis and hepatocellular carcinoma: a case report

**DOI:** 10.1186/1752-1947-5-160

**Published:** 2011-04-22

**Authors:** Mohammad Reza Ghadir, Mohammad Bagheri, Amir Hossein Ghanooni

**Affiliations:** 1 Qom University of Medical Sciences, Qom, Iran; 2 Tehran University of Medical Science, Digestive Disease Research Center, Tehran, Iran

## Abstract

**Introduction:**

Congenital hepatic fibrosis is an uncommon cause of portal hypertension. Despite the presence of portal hypertension, hepatocellular and renal function are usually well preserved. Congenital hepatic fibrosis is included in the group of congenital diseases of fibropolycystic disorders. These include a broad spectrum of clinical diseases which are usually accompanied by hepatic involvement.

**Case presentation:**

We report the case of a 27-year-old Iranian woman with congenital hepatic fibrosis leading to cirrhosis and subsequently hepatocellular carcinoma.

**Conclusion:**

Advanced cirrhosis was diagnosed and our patient was scheduled for liver transplantation. During preparation for transplant, a hepatic mass was discovered which was found to be hepatocellular carcinoma. Radiofrequency ablation was performed and our patient was referred for transplantation.

## Introduction

Congenital hepatic fibrosis (CHF) is an uncommon cause of portal hypertension. Despite the presence of portal hypertension, hepatocellular and renal function are usually well preserved. CHF is included in the group of congenital diseases of fibropolycystic disorders. These include a broad spectrum of clinical diseases which are usually accompanied by hepatic involvement.

Hepatocellular carcinoma (HCC) is a rare complication of CHF with only a few previous cases reported. We report the case of a 27-year-old woman with CHF who developed cirrhosis and HCC. Advanced cirrhosis was diagnosed and our patient was scheduled for liver transplantation. During preparation for transplant, a hepatic mass was discovered which was proved to be HCC. Radiofrequency ablation was performed and our patient was referred for transplantation.

## Case presentation

A 27-year-old Iranian woman was admitted to our hospital for evaluation of worsening hepatic function. She first came to medical attention at the age of 10 when hepatosplenomegaly was noted incidentally on a routine physical examination at another hospital, so she was admitted to undergo further examination. Her liver function was found to be within normal limits as was her renal function. Both her growth and development were normal for her age. However, hepatomegaly and splenomegaly were noted. A liver biopsy revealed proliferation of collagen fibers surrounding the portal area, a finding that was compatible with congenital hepatic fibrosis. Our patient had no history of hematemesis or tarry stool, but esophageal varices were detectable beginning at 10 years of age. Propranolol was started but she discontinued her medication after a couple of months despite medical advice.

**Figure 1:**
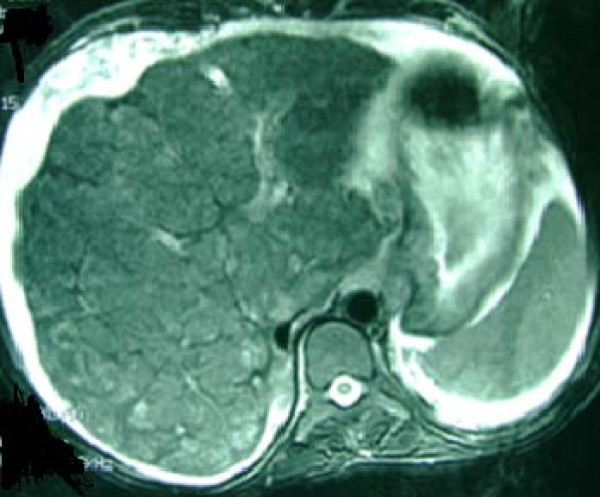
**Heterogeneous and cirrhotic liver in CT scan**.

Our patient was followed regularly without any complications such as abdominal pain, jaundice, hematemesis, tarry stool, or increases in liver enzymes. No history could be elicited of alcohol abuse or previous hepatitis. There was no family history of liver or kidney disease. Markers for hepatitis B and C were negative; urine and serum copper levels were normal; and serum auto-antibodies were negative. The liver size gradually decreased and portal pressure increased as documented by ultrasonography and computed tomography (CT) (Figure 1). Her portal vein diameter was 14 mm, and splenomegaly was observed. Grade 1 lower esophageal varices were reported during an upper gastrointestinal endoscopy. Our patient was put on a waiting list for liver transplantation.

**Figure 2:**
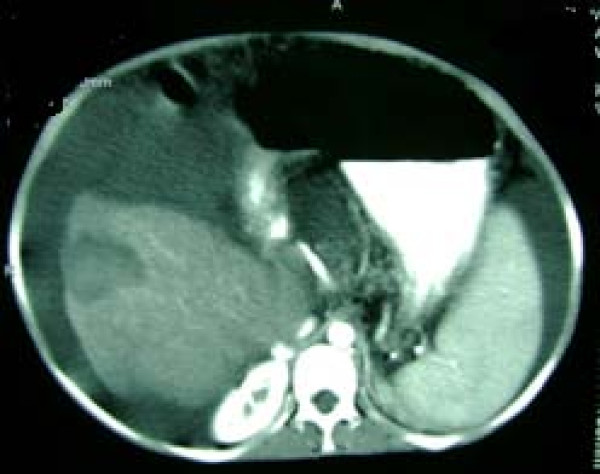
**a 59 × 39 mm mass in the right liver lobe**.

During preparation for transplantation, a 59 × 39 mm mass was observed in her right liver lobe, which was unnoted in previous evaluations (Figure 2). A percutaneous liver biopsy was reported to demonstrate HCC. Her serum alpha fetoprotein (AFP) level was 1900 IU/ml (normal < 6 IU/ml), serum glutamic oxaloacetic transaminase 72 IU/L, serum glutamic pyruvic transaminase 39 IU/L, alkaline phosphatise 120 IU/L, total bilirubin 3.2 mg/dL, direct bilirubin 2.3 mg/dL, albumin 2.7 g/dL, prothrombin time 16.1 seconds and her international normalized ratio 1.44.

**Figure 3:**
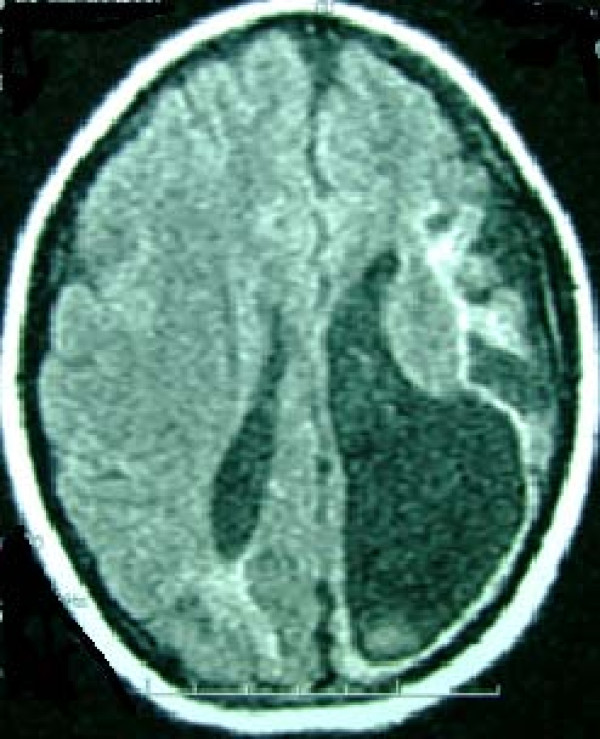
**Left hemispheric atrophy with severe dilation of the left ventricle**.

She was removed from the transplantation waiting list and was admitted for staging and planning treatment strategies. During admission, she developed abdominal pain, worsening abdominal distention and hepatic encephalopathy. Because of a decreased level of consciousness and seizure, a brain CT scan was done which revealed unexpected left hemispheric atrophy with severe dilation of her left ventricle (Figure 3). Proper management accompanied by frequent abdominal paracentesis led to partial improvement.

**Figure 4:**
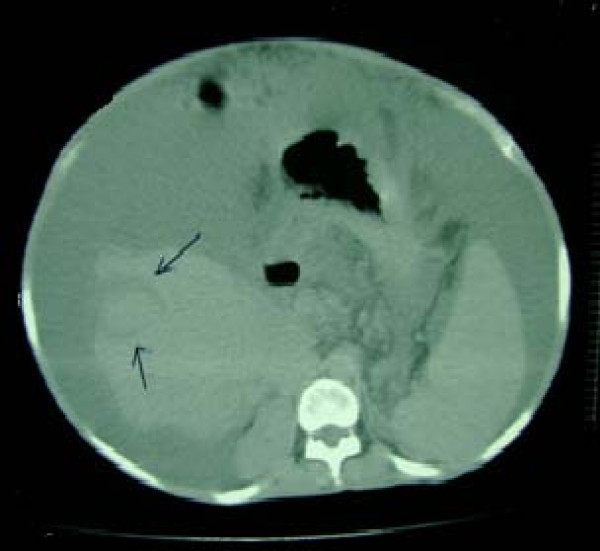
**Following radiofrequency ablation, tumor size declined**.

Following radiofrequency ablation, the tumor size declined to about 25 mm with central necrosis (Figure 4) and our patient was enrolled on to the liver transplantation waiting list again.

## Discussion

Congenital hepatic fibrosis is defined pathologically by bands of fibrous tissue within the liver, linking the portal area and containing multiple bile ductules. It occurs in association with a range of inherited disorders involving the kidneys. Although infantile-type polycystic kidney disease is usually an autosomal recessive disorder, our patient in the present report represents a sporadic case. Clinically, congenital hepatic fibrosis is characterized by portal hypertension with well-preserved liver function [1]. In adults, the disease is associated with two major risks: gastrointestinal hemorrhage caused by portal hypertension, and cholangitis due to bacterial infection of dilated intra-hepatic bile ducts. These episodes are sometimes fatal. The age at onset of symptoms may be as young as three to six months [2,3]. The usual age of presentation is between 1.8 and 14 years [4]. Tubular renal dysfunction is present in less than 10% of patients. This usually has no clinical manifestation, except in the presence of portal hypertension or cholangitis [5]. Ockendens divided his patients into four age groups: perinatal, neonatal, infantile, and adolescent. The highest prevalence of renal involvement was noted in the perinatal group and lowest in the adolescent group [6]. The highest prevalence of cholangitis was reported in the adolescent group.

Our patient was in the adolescent group, but no episodes of cholangitis were found. Our patient had no history of fever and jaundice, nor any evidence of cholangitis; however, she developed cirrhosis and hepatic failure which necessitated liver transplantation. Therefore, we could not explain the cause of cirrhosis by repeated cholangitis.

CHF is closely associated with neonatal polycystic kidney disease and dilation of the intrahepatic biliary tree (Caroli's Disease). Other disorders associated with CHF are medullary sponge kidney, Ivemark familial dysplasia, Meckel syndrome, vaginal atresia, and with less prevalence in adults, polycystic kidney, and tuberous sclerosis [7].

Although these conditions had been ruled out in our patient, the severe dilation of her brain ventricles, especially in her left lateral ventricle due to left hemispheric atrophy, remains of special interest. Our case may add another new category to these known clinical types of CHF, which include cirrhosis, HCC, and unusual extrahepatic manifestations in the brain. However, more cases should be studied before a definite conclusion can be made.

A diagnosis of CHF is suggested when normal hepatocellular function is associated with hypersplenism and increased levels of alkaline phosphatase and gamma glutamyl transferase [8]. Definite diagnosis is by liver biopsy [4]. Prognosis is dependent on the degree of portal hypertension, the signs and symptoms of which can be decreased by surgical shunts [1]. As mentioned, CHF distorts the hepatic structure without any effect on hepatocellular function, so levels of liver enzymes are generally within normal ranges. In cirrhosis, in contrast to CHF, hepatocellular injury occurs and the abnormal level of liver enzymes is a distinctive feature [9]. The question is whether CHF could be a precursor to liver cirrhosis? In the search for diverse causes of liver cirrhosis, CHF has not previously been considered [10]. This is despite a few cases reporting an association between CHF and cirrhosis [11].

## Conclusion

We propose that there be a high index of suspicion for the development of HCC in patients with CHF, particularly in those presenting with jaundice and cirrhosis. Screening could be undertaken with serum AFP measurements and hepatic imaging studies, using ultrasound, CT, or magnetic resonance imaging.

## Abbreviations

AFP: alpha fetoprotein; CHF:congenital hepatic fibrosis; CT: computed tomography; HCC: hepatocellolar carcinoma.

## Consent

Written informed consent was obtained from the patient for publication of this case report and any accompanying images. A copy of the written consent is available for review by the Editor-in-Chief of this journal.

## Competing interests

The authors declare that they have no competing interests.

## Authors' contributions

MRG had the initial idea for this work and was responsible for writing the manuscript. AHG handled the patient data acquisition and the literature research. MB contributed to the editing of the manuscript. MRG was responsible for creating the figure files. MRG contributed to the editing and writing of the manuscript. All authors read and approved the final version of the manuscript.
